# Sex Matters: A Comprehensive Comparison of Female and Male Hearts

**DOI:** 10.3389/fphys.2022.831179

**Published:** 2022-03-22

**Authors:** Sarah R. St. Pierre, Mathias Peirlinck, Ellen Kuhl

**Affiliations:** ^1^Department of Mechanical Engineering, Stanford University, Stanford, CA, United States; ^2^Department of Biomechanical Engineering, Delft University of Technology, Delft, Netherlands; ^3^Department of Biomedical Engineering, Erasmus MC, Rotterdam, Netherlands

**Keywords:** sex differences, cardiac remodeling, athlete's heart, dilated cardiomyopathy, hypertrophic cardiomyopathy, eccentric hypertrophy, concentric hypertrophy, sex-specific diagnostics

## Abstract

Cardiovascular disease in women remains under-diagnosed and under-treated. Recent studies suggest that this is caused, at least in part, by the lack of sex-specific diagnostic criteria. While it is widely recognized that the female heart is smaller than the male heart, it has long been ignored that it also has a different microstructural architecture. This has severe implications on a multitude of cardiac parameters. Here, we systematically review and compare geometric, functional, and structural parameters of female and male hearts, both in the healthy population and in athletes. Our study finds that, compared to the male heart, the female heart has a larger ejection fraction and beats at a faster rate but generates a smaller cardiac output. It has a lower blood pressure but produces universally larger contractile strains. Critically, allometric scaling, e.g., by lean body mass, reduces but does not completely eliminate the sex differences between female and male hearts. Our results suggest that the sex differences in cardiac form and function are too complex to be ignored: the female heart is not just a small version of the male heart. When using similar diagnostic criteria for female and male hearts, cardiac disease in women is frequently overlooked by routine exams, and it is diagnosed later and with more severe symptoms than in men. Clearly, there is an urgent need to better understand the female heart and design sex-specific diagnostic criteria that will allow us to diagnose cardiac disease in women equally as early, robustly, and reliably as in men.

**Systematic Review Registration:**

https://livingmatter.stanford.edu/.

## 1. Introduction

The adult human heart is about the size of a fist, and it weighs on the order of 300 g (Gray, 1878). This makes it more than three orders of magnitude larger than the heart of a mouse (Slawson et al., 1998) and more than three orders of magnitude smaller than the heart of a whale (Race et al., 1959). While the dimensions of the human heart are often reported as fixed values, its size and weight are neither constant throughout life, nor are they similar for women and men (Molina and DiMaio, 2012, 2015). The human heart is an amazing and living system that continuously adapts to meet our body's demands and supplies all our organs with sufficient oxygen and nutrition (Humphrey, 2002).

The purpose of the heart is to circulate blood from venous return. This naturally limits cardiac output and is largely influenced by the metabolic rate of our peripheral tissues (Wolff, 2008). Traditionally, the heart has been considered permissive, meaning it does not regulate its own output (Carlson and Johnson, 1948). Since metabolism is difficult and cumbersome to measure, for decades, scientists have scaled the heart relative to body size, e.g., to overall body height, weight, surface area, or lean body mass, as a proxy for metabolic demand. When adjusted by lean body mass, neither resting metabolic rate (Buchholz et al., 2001) nor basal metabolic rate (Cunningham, 1980) are different between men and women. However, the absolute performance of our heart, the cardiac output, naturally varies significantly with age, sex, and, most notably, with exercise. Both exercise and disease can cause cardiac remodeling, a process by which the heart adapts to mechanical stimuli by increasing cardiomyocyte length, as in dilated cardiomyopathy, or width, as in hypertrophic cardiomyopathy (Opie, 2003). This means that for dilated cardiomyopathy, the chambers of the heart become enlarged, while in hypertrophic cardiomyopathy, the walls of the ventricles thicken (Genet et al., 2016). Understanding these variations is significant when optimizing cardiac function, diagnosing cardiac disease, or designing cardiac treatment.

**Figure 1:**
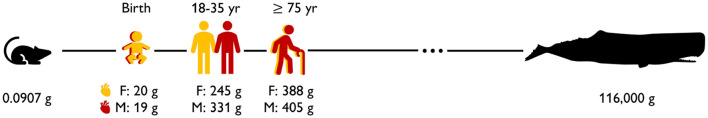
Sex differences in the healthy human heart across life. At birth, the female heart is +5% larger than the male heart (Altman and Dittmer, 1962); during adulthood, the female heart becomes −26% smaller than the male heart (Molina and DiMaio, 2012, 2015); in the elderly, differences become less pronounced and the female heart is −4% smaller than the male heart (Sheikhazadi et al., 2010). Mouse (Slawson et al., 1998) and whale (Race et al., 1959) hearts are shown for comparison.

Figure 1 illustrates the sex differences in the healthy human heart across life. Strikingly, at birth, the human heart has less than a tenth of its maximum adult mass. As we age, the total number of heart muscle cells remains the same but the cells themselves increase in volume. This causes the heart to grow (Bergmann et al., 2015). With 20 g, the newborn female heart is +5% larger than the male heart with only 19 g (Altman and Dittmer, 1962). While female and male hearts remain comparable in size at a younger age, the male heart grows significantly faster during puberty (de Simone et al., 1995). This results in a notable mismatch in mass and size as we reach adulthood. On average, the mass of the adult female heart ranges from 230 to 280 g and is about −26% lighter than the male heart, which varies from 280 to 340 g (Gray, 1878). For both women and men, the mass of the heart continues to increase with age, and female hearts remain consistently smaller than male hearts (Molina and DiMaio, 2012, 2015). In the elderly, female hearts, with an average mass of 388 g, are about −4% smaller than male hearts, with an average mass of 405 g (Sheikhazadi et al., 2010).

The female and male hearts do not only differ in mass and size but also display a myriad of functional, structural, genetic, and hormonal differences: women have a higher resting heart rate than men, but their hearts take a long time from contraction to relaxation. This is a result of the action of testosterone during ventricular repolarization (James et al., 2007), and we can observe it through longer QT intervals in the electrocardiogram. This puts women at greater risk of drug-induced arrhythmias (Peirlinck et al., 2021a). Increasing evidence suggests that both progesterone and testosterone are protective against arrhythmias, while estrogen may increase susceptibility to rhythm disorders (Yang and Clancy, 2010). From a functional point of view, sex hormones are involved in the regulation of calcium homeostasis, which leads to sex differences in the cardiac excitation-contraction coupling pathway (Parks and Howlett, 2013). With regard to cardiac autonomic function, women have more vagal control over sympathetic responsiveness for cardiac function than men (Dart et al., 2002). Myocardial metabolism is directly linked to cardiac function; sex differences exist in myocardial oxygen consumption and glucose utilization (Wittnich et al., 2013). Estrogen has been shown to decrease glucose utilization, meaning fatty acid oxidation is more responsible for energy production in women than in men (Wittnich et al., 2013). This may explain the cardioprotective effect of estrogen, provided the myocardium is well-oxygenated (Wittnich et al., 2013). Recent studies have also found sex differences in metabolic adaptation among endurance athletes, with women and men reducing body fat, increasing oxygen uptake, and increasing left ventricular mass after different lengths of training and to different extents (Regitz-Zagrosek and Kararigas, 2017).

We hypothesize that these sex-specific differences collectively result in a different cardiac performance for women and men and in a sex-specific adaptation to physiological and pathological overload. The objective of this manuscript is to review sex-specific differences in cardiac form and function, discuss where these differences matter, and provide recommendations on how to address these discrepancies. A better understanding of sex differences in the human heart is critical to designing sex-specific training plans that improve human performance and prevent cardiac injuries in athletes and to establish sex-specific diagnostic criteria that accurately identify the cardiac disease and improve cardiac health.

## 2. Sex Differences in the Healthy Heart

In this section, we summarize and discuss geometric and functional sex differences in healthy adult human hearts.

### 2.1. Sex Differences in Healthy Heart Geometry

**Figure 2:**
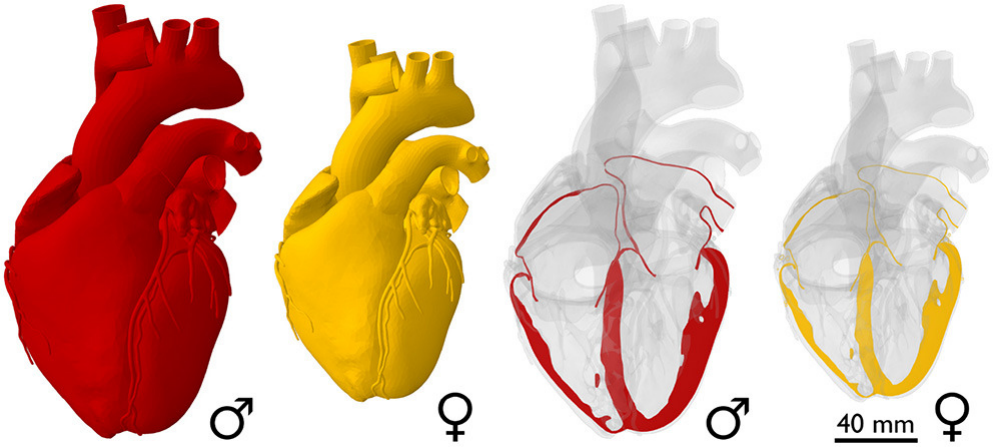
Sex differences in healthy heart size. The whole-heart view compares an average male human heart (Zygote Media Group Inc., 2014), shown in red, to an isometrically scaled down female heart, which is −26% smaller in mass. Based on isometric scaling, the female ventricular wall thickness and its ventricular and atrial diameters would be −9.0% smaller than their male counterparts.

**The female heart is one-fourth smaller than the male heart**. Figure 2 illustrates the sex-specific size differences of the healthy human heart. As a first approximation, these images assume an isometric scaling between male and female hearts. With a mass of 245 g, the female human heart weighs on average 26% less than the male heart with a mass of 331 g (Molina and DiMaio, 2012, 2015). If we assume an isometric scaling with -26%, the female wall thickness and its ventricular and atrial diameters would be approximately (1.00−0.26)^1/3^ = 0.90 times the size of their male counterparts, meaning they would be −9.0% smaller. It seems intuitive to ask whether geometric features scale proportionally between healthy male and female human hearts and, if so, how can we identify appropriate scaling factors?

**Scaling by lean body mass can reduce but not eliminate sex differences in mass**. Table 1 summarizes the main geometric differences between healthy male and female hearts. All features, except the body fat percentage, heart rate, and right ventricular ejection fraction, are significantly smaller in women than in men, on average by about one-fourth. To eliminate these differences, various studies have proposed different scaling metrics (Dewey et al., 2008). A recent study found that allometric scaling by body surface area does not eliminate sex differences between men and women (Petersen et al., 2017). In contrast, allometric scaling by lean body mass or fat free mass only accounts for the active mass of the body, not the fat, and seems to be a more appropriate approach (Giraldeau et al., 2015). Healthy young adult women have a lean body mass of 36.5 kg compared to men of 56.7 kg (Zhu et al., 2014). Based on the −36% smaller lean body mass in women, we would expect a whole heart mass difference of −36%. Indeed, this correlation holds for the left ventricular mass difference of −34% (Vasan et al., 1997) between men and women. However, it overestimates the right ventricular mass difference of −25% (Sandstede et al., 2000) and also the whole heart mass difference of −26% recorded *via* autopsy after sudden, traumatic death in healthy 18 to 35-year-old men and women (Molina and DiMaio, 2012, 2015). This suggests that scaling by lean body mass can help reduce, but not entirely eliminate, sex differences in cardiac geometry.

**Table 1:**
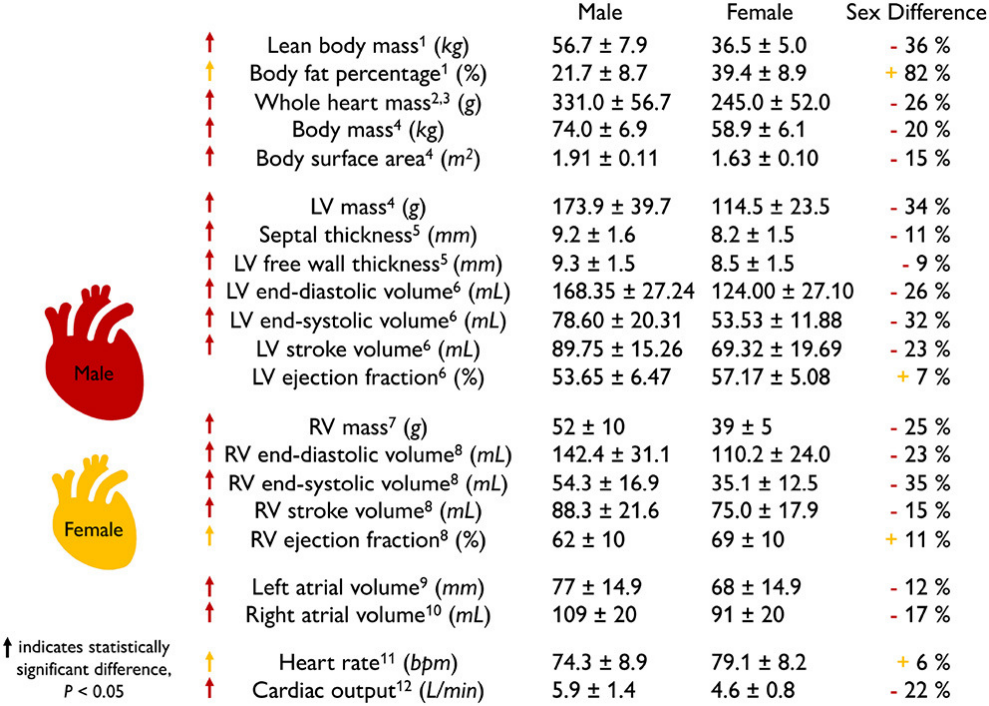
Sex differences in healthy heart geometry and function.

*The male heart has greater mass, volume, and cardiac output than the female heart, while the female heart has slightly greater ejection fractions and heart rate. Male and female characteristics and sex differences are reported in percent, with the male heart as the baseline. Negative values in red indicate that the female parameter is smaller, positive values in yellow indicate that the female parameter is larger; arrows indicate reported statistically significant differences; quantitative data are represented as mean absolute value ± SD; [1] Zhu et al. (2014); [2] Molina and DiMaio (2012); [3] Molina and DiMaio (2015); [4] Vasan et al. (1997); [5] Kou et al. (2014); [6] Rutkowski et al. (2020); [7] Sandstede et al. (2000); [8] Tandri et al. (2006) [9] Maceira et al. (2010); [10] Maceira et al. (2013); [11] Ramaekers et al. (1998); [12] Argiento et al. (2012)*.

**The female heart is not just a small version of the male heart**. Table 1 quantifies important geometric sex differences in the healthy human heart including whole heart mass, left and right ventricular mass, and wall thickness. The absolute numbers confirm that a simple isometric scaling by lean body mass (Giraldeau et al., 2015) or whole heart mass similar to Figure 2 provides a good first approximation. However, it is obvious that some geometric features do not scale isometrically. For example, the female left ventricular mass is −34% smaller than the male mass (Vasan et al., 1997), while the right ventricular mass is only −25% smaller (Sandstede et al., 2000). If we perform an isometric scaling of only the left ventricle by −34%, the female ventricular wall would be (1.00 − 0.34)^1/3^ = 0.87 times the size of the male wall, meaning it would be −13% thinner. This difference is larger than both the observed difference in septal thickness of −11% and in left ventricular free wall thickness of −9% (Kou et al., 2014). These simple estimates underscore, once more, that male and female hearts do not simply scale isometrically in cardiac dimensions and that the female heart is not just a small version of the male heart. It seems natural to ask whether and how these disproportional sex differences in chamber size and wall thickness translate into differences in cardiac function between healthy male and female hearts.

### 2.2. Sex Differences in Healthy Heart Function

**The female heart has a smaller cardiac output than the male heart**. Table 1 highlights functional sex differences in the human heart including left and right ventricular volumes, ejection fraction, heart rate, and cardiac output. With end-diastolic and end-systolic volumes of 124 and 53.53 ml, the female heart has a -23% smaller stroke volume of 69.32 ml than the male heart with end-diastolic and end-systolic volumes of 168.35 and 78.60 ml and a stroke volume of 89.75 ml (Rutkowski et al., 2020). Interestingly, the female heart attempts to compensate for this difference by a +6% larger heart rate with 79.1 bpm compared to the male heart with 74.3 bpm (Ramaekers et al., 1998). Nonetheless, the cardiac output remains consistently smaller in women than in men, 5.6 vs. 6.7 L/min, resulting in −16%, and 4.6 vs. 5.9 L/min, resulting in −22% (Argiento et al., 2012) as reported in Table 1. Interestingly, when scaled by the female and male lean body mass of 36.5 kg and 56.7 kg, the female heart with 0.126 L/[kg·min] has a 21% larger score than the male heart with 0.104 L/[kg·min]. A natural question to ask is how sex differences in cardiac output translate into differences in ejection fraction.

**The female heart has a larger ejection fraction than the male heart**. The ejection fraction is the ratio between stroke volume and end-diastolic volume and is an important indicator of ventricular efficiency. The female-to-male end-diastolic volume differs by −26 and −23% for the left and right ventricles, which is in close agreement with the female-to-male whole heart mass difference of −26%. At the same time, the female-to-male end-systolic volume differs by −32 and −35% for the left and right ventricles. These numbers suggest that the female systolic heart is disproportionally smaller than the male systolic heart. Interestingly, these volume differences result in a larger ejection fraction of +7 and +11% for the female left and right ventricles compared to their male counterparts. This raises the question of whether and how sex differences in ejection fraction on the whole organ scale translate into differences in myocardial strains on the tissue and cellular scale.

### 2.3. Sex Differences in Healthy Myocardial Strains

**The female heart has 10–14% larger contractility than the male heart**. Table 2 summarizes the sex differences in strains and strain rates in the healthy human heart. Interestingly, most reported strains are significantly larger in the female heart with circumferential strains by +11 and +10% and longitudinal strains by +14 and +13% in the left (Lawton et al., 2011) and right (Lakatos et al., 2020) ventricles. Strain rates are more cumbersome to measure and their sex differences vary between +13 and +49%, but all female strain rates are consistently larger than male (Rutkowski et al., 2020). We can correlate the reported values in Tables 1, 2 through a simple back-of-the-envelope half-sphere calculation: based on the reported left ventricular end-diastolic and end-systolic volumes of 168.35 and 78.6 ml for the male and 124 and 53.53 ml for the female heart in Table 1, this estimate would predict male and female left ventricular strains of -22 and -24% and a female-to-male left strain difference of +9%. This estimate is consistent with the *in vivo* measured differences of +11% circumferentially and +14% longitudinally (Lawton et al., 2011) in Table 2. These observations raise the question to which extent differences in myocardial microstructure and myocyte ultrastructure can help explain the consistently larger negative strains and contractility of the female heart.

**Table 2:**
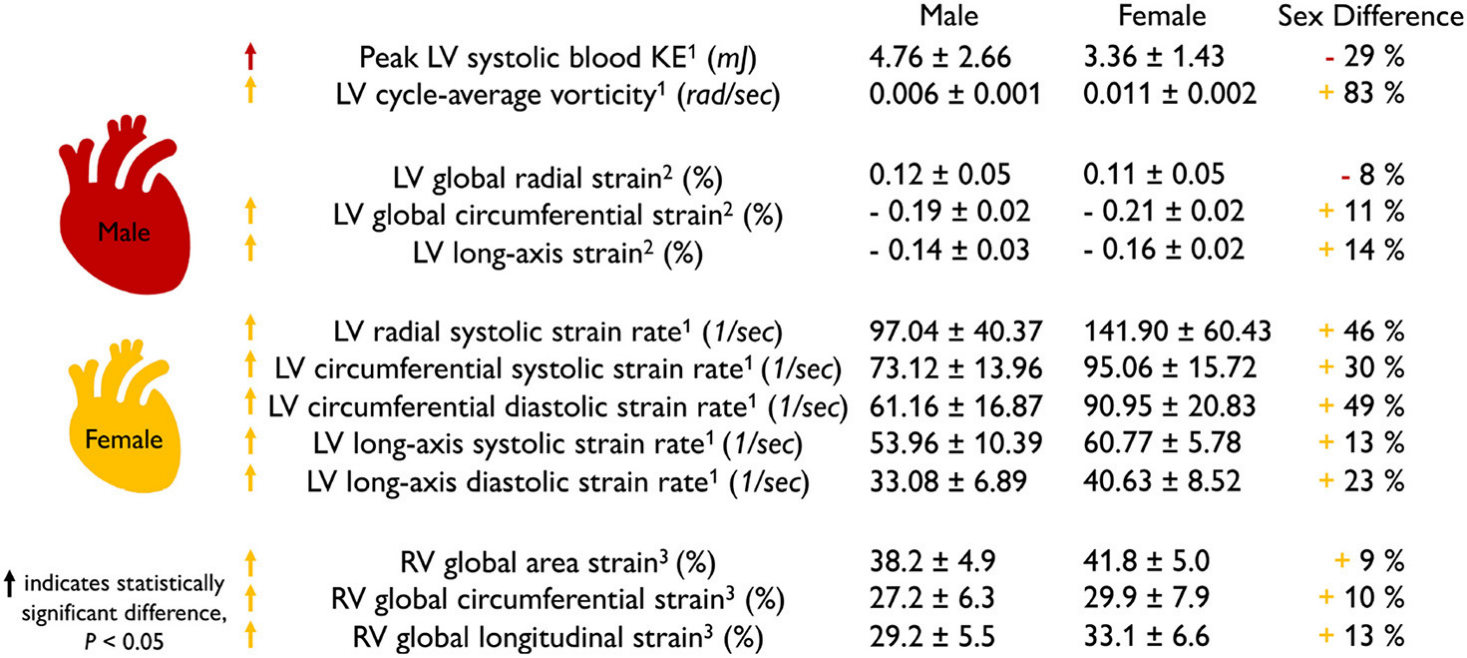
Sex differences in healthy myocardial strains.

*Sex differences between female and male flow, strain, and strain rate, with a male as the baseline. While the male heart has greater systolic blood kinetic energy, the female heart exhibits strains that are significantly larger than the male for radial, circumferential, and longitudinal axes and both left and right ventricular chambers of the heart. Negative values in red indicate that the female parameter is smaller, positive values in yellow indicate that the female parameter is larger; arrows indicate reported statistically significant differences; quantitative data are represented as mean absolute value ± SD; [1] Rutkowski et al. (2020); [2] Lawton et al. (2011); [3] Lakatos et al. (2020)*.

### 2.4. Sex Differences in Healthy Blood Pressure

**The female blood pressure is lower than the male blood pressure**. Table 3 summarizes the sex differences in healthy blood pressure for both the male and the female heart for three different measurement techniques, transseptal catheters, tonometry, and sphygmomanometry. Both the aortic blood pressure, measured by tonometry (Doonan et al., 2013), and the brachial blood pressure, measured by sphygomanometry (Vasan et al., 1997; Avolio et al., 2018), show significantly lower values for the female than for the male heart. Interestingly, the differences in systolic pressure of −9 and −6% are slightly larger than the difference in diastolic pressure of −6 and −5%. Measuring the blood pressure in the left ventricle directly is an invasive procedure that can be performed using transseptal catheters. The reported female left ventricular catheter pressures of 131 mmHg at peak systole and 9 mmHg at end diastole are −2 and −31% smaller than their male values of 133 and 13 mmHg (Villari et al., 1995). Taken together, for all three measurement techniques, the female blood pressure is consistently lower than the male blood pressure. This seems to be a natural consequence of differences in both myocardial composition and wall thickness, since the female heart muscle is 9% thinner than its male counterpart. Smaller blood pressure in women could be a natural adaptation to a smaller systemic resistance. This is not necessarily concerning; it could simply be the result of differences in average height between men and women, which, for the example of the United States with 175.3 and 161.3 cm, would result in a −8% difference (Fryar et al., 2021).

**Table 3:**
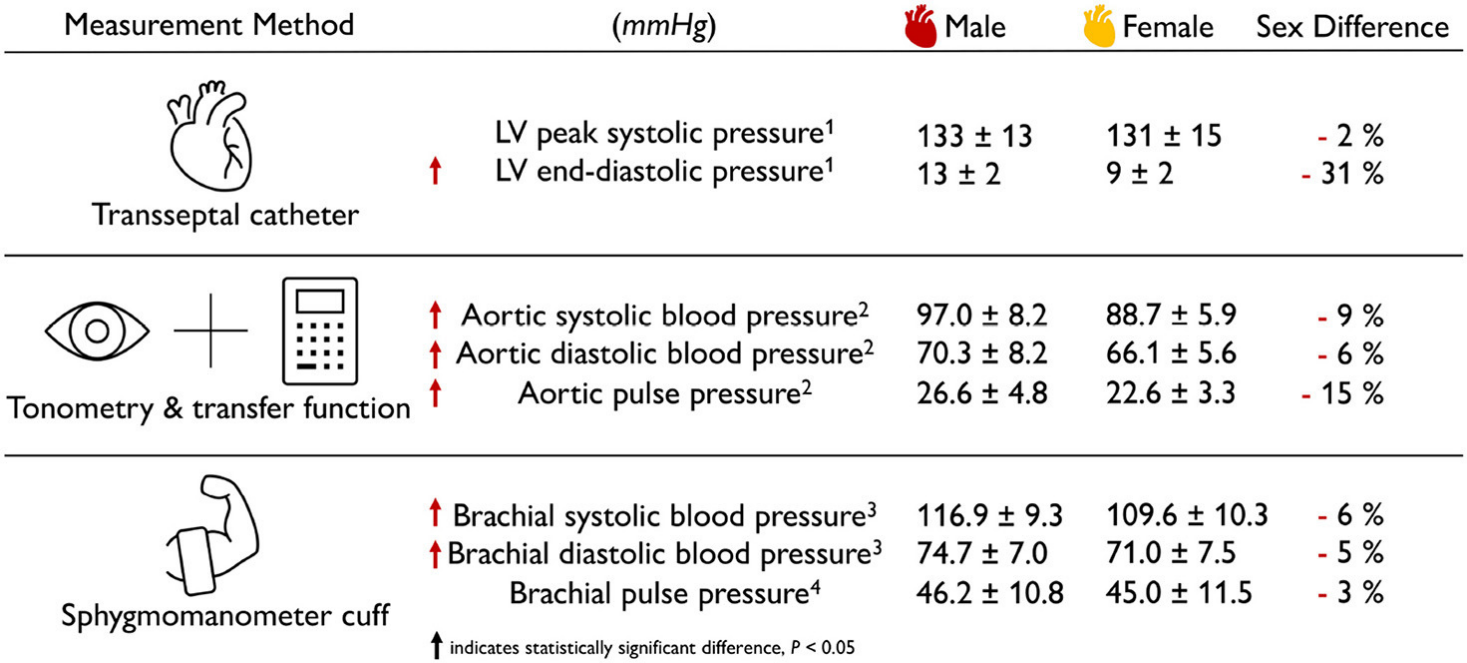
Sex differences in healthy blood pressure.

*Sex differences between female and male blood pressures in percent, with the male heart as the baseline. With the use of the transseptal catheter, the most invasive blood pressure measuring technique, the male heart is shown to have significantly greater end-diastolic pressure than the female heart, there was no significant difference for systolic pressure. With less invasive techniques, the differences between male and female blood pressure were between 5–15%, with the male pressure always greater than the female. Negative values in red indicate that the female parameter is smaller; arrows indicate reported statistically significant differences; quantitative data are represented as mean absolute value ± SD; [1] Villari et al. (1995); [2] Doonan et al. (2013); [3] Vasan et al. (1997); [4] Avolio et al. (2018)*.

**Allometric scaling by lean body mass eliminates sex differences in cardiac reserve**. The cardiac reserve is the difference between the maximum cardiac power output at exercise and the cardiac power output at rest. As such, it is an important health indicator for several medical conditions. Cardiac power output is the product of the arterial pressure reported in Table 3, the cardiac output reported in Table 1, and a conversion factor to translate the units into watts. A study of healthy untrained individuals recorded sex-specific mean maximum cardiac power outputs at exercise of 5.3 and 4.0 W, mean cardiac power outputs at rest of 1.0 and 0.8 W, and cardiac reserves of 4.3 and 3.2 W for men and women (Chantler et al., 2005). When scaled allometrically by body surface area or body mass, the sex differences for all three metrics remained statistically different. However, when scaled by lean body mass, the sex differences in cardiac reserve became statistically insignificant, while differences in cardiac power output remained. The lean body mass scaling coefficients varied between 0.71, 0.47, and 0.79 for maximal exercise, rest, and reserve. These discrepancies highlight that there is no single unified scaling parameter that could explain the geometric and functional sex differences in Tables 1–3.

**The female wall stiffness is different from the male stiffness**. We can use the law of Laplace to estimate the wall stiffness upon passive filling from simple back-of-the-envelope calculations. We use the reported male and female left ventricular free wall thicknesses of 9.3 and 8.5 mm (Kou et al., 2014), end-diastolic volumes of 168.35 and 124.00 ml (Rutkowski et al., 2020), end-systolic volumes of 78.60 and 53.53 ml (Rutkowski et al., 2020), and diastolic pressures of 13 and 9 mmHg (Villari et al., 1995). Using the simple kinematics of an inflated sphere, we estimate the male and female end-systolic radii to 26.6 and 23.4 mm and the end-distolic radii to 34.3 and 30.9 mm, resulting in male and female wall strains of 0.29 and 0.32. Using the law of Laplace, we estimate the male and female wall stresses as the product of the diastolic pressures 13 and 9 mmHg and the end-systolic radii 26.6 and 23.4 mm, divided by twice the wall thickness, 2·9.3 and 2·8.5 mm, resulting in male and female wall stresses of 0.00247 and 0.00165 N/mm^2^. Using Hooke's law, we estimate the passive wall stiffness as the ratio between stresses and strains resulting in male and female wall stiffnesses of 8.55 and 5.09 kPa. While these absolute values are likely overestimates of the true stiffness because of the idealized linear kinematics, linear law of Laplace, and linear Hooke's law, they provide valuable estimates to compare the mechanics of the male and female heart: This simple back-of-the-envelope calculation suggests that, upon passive filling, female myocardial strains are +12% larger, stresses are -33% smaller, and stiffness is -40% smaller than their male counterparts. In contrast, the literature reports that female hearts are generally stiffer than male (Redfield et al., 2005; Beale et al., 2018; Regitz-Zagrosek, 2020) and that stiffness increases with aging more notably in women than in men. It will be important to understand differences in myocardial microstructure and cardiomyocyte ultrastructure to explain and understand sex-specific differences in ventricular wall stiffness.

**The female myocardium is richer in cardiomyocytes than the male myocardium**. A recent cardiac MRI radiomics study of 6,095 male and 8,807 female healthy hearts found that the female myocardium was less dimmer and more texturally complex than the male, and the ventricles were smaller and less elongated (Raisi-Estabragh et al., 2021). Interestingly, the extracellular volume fraction, used to quantify interstitial myocardial fibrosis, is greater in women than men Liu et al. (2013); Roy et al. (2017). A higher extracellular volume fraction in women could be interpreted as more dispersed fibrosis, and if this is the case, this would point to a stiffer female heart. In general, male and female hearts have similar cell distributions except for the percentage of ventricular cardiomyocytes. A recent study found that the female heart, made up of 56 ± 9 % ventricular cardiomyocytes, has +19.2% more cardiac muscle cells than the male heart with 47 ± 11 % (Litviňuková et al., 2020). While this difference is unexpected and needs to be confirmed by further studies, it might explain the +10 to +14% larger contractile strains in female hearts compared to male hearts.

## 3. Sex Differences in the Athlete's Heart

The athlete's heart is a condition of extreme physiological adaptation to pressure and volume overload in the hearts of individuals who participate in intense athletic exercise. In this section, similar to the healthy heart in Section 2, we summarize and discuss geometric and functional sex differences in athletes' hearts.

### 3.1. Sex Differences in Athlete's Heart Geometry

**The hearts of male and female athletes are larger than those of non-athletes**. Table 4 summarizes the main geometric features of male and female athletes' hearts. In comparison to non-athletes' hearts in Table 1, all left and right ventricular values, male and female, are larger in athletes than in non-athletes, except for the right ventricular mass and ejection fraction (Csecs et al., 2020). For example, the left ventricular mass in male and female athletes is 207.8 and 143.9 g (D'Ascenzi et al., 2020), which is 20 and 26% larger, compared to 173.9 and 114.5 g in non-athletes (Vasan et al., 1997). Interestingly, despite these absolute variations, the relative sex differences between non-athletes in Table 1 and athletes in Table 4 are quite similar. The female heart is consistently smaller in left and right ventricular mass (Csecs et al., 2020; D'Ascenzi et al., 2020), end-diastolic and end-systolic volumes (Csecs et al., 2020), and stroke volume (Giraldeau et al., 2015; Csecs et al., 2020) both in non-athletes and athletes, all on the order of one-fourth. In contrast, the female heart is consistently larger in left and right ventricular ejection fraction both in non-athletes (Tandri et al., 2006; Rutkowski et al., 2020) and athletes (Csecs et al., 2020; D'Ascenzi et al., 2020), with sex differences of +7, +11, +3, and +5%. Since athletes tend to have different lean body mass than non-athletes, a natural question to ask is to which extent discrepancies in cardiac dimensions would disappear when scaled by lean body mass.

**Table 4:**
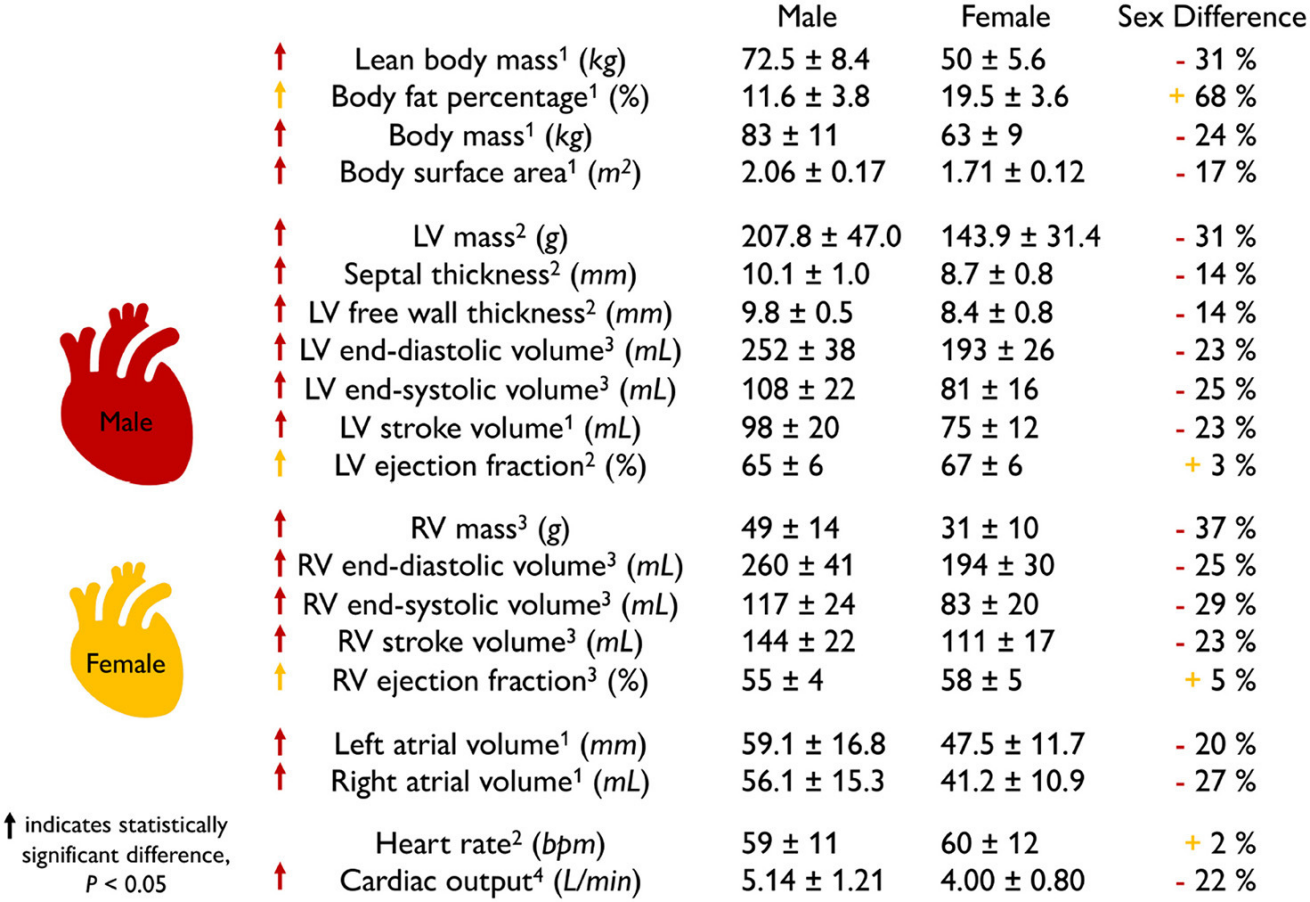
Sex differences in athlete's heart geometry and function.

*Similar to the healthy population, the male athlete's heart has greater heart mass, volume, and cardiac output than the female athlete's hearts, while the female athlete's heart has slightly greater ejection fractions. Male and female characteristics and sex difference are reported in percent, with the male heart as the baseline. Negative values in red indicate that the female parameter is smaller; arrows indicate reported statistically significant differences; quantitative data are represented as mean absolute value ± SD; [1] Giraldeau et al. (2015); [2] D'Ascenzi et al. (2020); [3] Csecs et al. (2020); [4] Cote et al. (2015)*.

**Scaling by lean body mass eliminates sex and athletic differences in left ventricular mass**. Table 4 summarizes cardiac geometries, lean body mass, and body fat percentage of male and female athletes. To no surprise, the left ventricular mass differs significantly between male and female athletes with 207.8 and 143.9 g (D'Ascenzi et al., 2020) compared to male and female non-athletes with 173.9 and 114.5 g (Vasan et al., 1997). Scaling by the lean body mass of 72.5 and 50 kg for male and female athletes (Giraldeau et al., 2015) and of 56.7 and 36.5 kg for non-athletes (Zhu et al., 2014), significantly reduces differences between athletes and non-athletes and entirely eliminates differences between male and female hearts: the left-ventricular-mass-to-lean-body-mass index is 0.29% in athletes and 0.31% in non-athletes, and is identical for men and women. Similar to the non-athlete heart, scaling by lean body mass is less successful for the right ventricle: the right-ventricular-mass-to-lean-body-mass index varies widely from 0.68 and 0.62% in male and female athletes to 0.92 and 1.07% in male and female non-athletes. The observation that scaling by lean body mass works well for the left ventricle, but not universally for all geometric features, underscores once more that the female heart is not just a small version of the male heart, neither for non-athletic nor for athletic individuals.

**The female athlete's heart is not just a small version of the male athlete's heart**. Table 1 quantifies important geometric sex differences in athletes' hearts including ventricular mass and volumes, and wall thicknesses. Similar to the non-athlete heart, a simple isometric scaling by lean body mass (Giraldeau et al., 2015) or whole heart mass similar to Figure 2 could provide a good first approximation to eliminate size differences. However, if we perform an isometric scaling of only the left ventricle by -31%, the female ventricular wall would be (1.00 − 0.31)^1/3^ = 0.88 times the size of the male wall, meaning it would be -12 % thinner. Comparing this estimated difference of -12 % to the measured difference of -14 % in both septal thickness and left ventricular free wall thickness (D'Ascenzi et al., 2020) reveals that female athletes have disproportionally thinner hearts than male athletes and these sex differences cannot be explained by isometric scaling alone. In a study of 1,083 elite athletes, 40% of whom were female, no female athlete had a relative wall thickness greater than 12 mm, or a left ventricular end-diastolic diameter greater than 54 mm, while some of the male athletes did (Finocchiaro et al., 2017). Male and female athletes participated in similar hours of exercise per week and were subdivided into dynamic, static, and mixed groups based on Mitchell's classifications, refer to **Figure 5**. Strikingly, only 7% of the female athletes in this study developed concentric remodeling, while 12% of the male athletes did, indicating that concentric hypertrophy in elite female athletes is more likely a marker of disease than in male athletes (Finocchiaro et al., 2017). A study of 360 female and 360 age- and sport-matched male Olympic athletes found virtually no left ventricular wall thickening in female athletes, while some male athletes did show concentric remodeling or hypertrophy (D'Ascenzi et al., 2020). These sex-specific differences in cardiac adaptation among elite athletes may help explain the 10:1 male-to-female ratio in sports-related sudden cardiac deaths (Colombo and Finocchiaro, 2018).

### 3.2. Sex Differences in Athlete's Heart Function

**Sex differences in cardiac geometry and function remain present but are not magnified, in athletes**. Table 4 highlights functional sex differences in the human heart including left and right ventricular volumes, ejection fraction, heart rate, and cardiac output. With end-diastolic and end-systolic left ventricular volumes of 193 and 81 ml the female heart has a -23% smaller stroke volume of 75 ml, than the male heart with end-diastolic and end-systolic volumes of 252 and 108 ml and a stroke volume of 98 ml (Csecs et al., 2020). Interestingly, when scaled by the female and male lean body mass of 50.0 and 72.5 kg (Giraldeau et al., 2015), the relative female athlete's stroke volume of 1.50 ml/kg would be 11% larger than the relative male athlete's stroke volume of 1.35 ml/kg. While the left ventricular stroke volumes of female and male athletes of 75 and 98 ml (Csecs et al., 2020) are 8 and 9% larger than in non-athletes with 69 and 90 ml (Rutkowski et al., 2020), the relative difference between female and male hearts remains comparable with -23% between athletes and -23% between non-athletes. These observations suggest that the female heart adapts to intense exercise similar to the male heart, and relative sex differences remain present but are not magnified in athletes. With the reported sex differences on the order of 20–30% in Table 4, an interesting question to ask is how do these geometric and functional differences translate into differences in athletic performance. Or, in other words, if geometric and functional features scale universally on the order of 20–30% between female and male athletes, why is the difference between the current female and male marathon world records, 2:14:04 and 2:01:39, respectively, only 10.2% (Douglas, 2021)?

**Lean body mass scaling with different scaling factors eliminates sex differences in athletes' hearts**. Similar to healthy adults, athletic young adult women have 31% less lean body mass than men (Giraldeau et al., 2015). From moderately exercising people to elite athletes, numerous studies have tried to explain differences in cardiac mass, dimension, and function by scaling to either lean body mass, fat free mass, or a combination of both (Hutchinson et al., 1991; Giraldeau et al., 2015; Bassareo and Crisafulli, 2020). Yet, the reported scaling factors can vary significantly: for left ventricular mass, left ventricular atrial volume, left ventricular end-diastolic volume, stroke volume, and wall thickness, studies report scaling factors of 1.00, 0.70, 0.70, 0.70, and 0.33 (Giraldeau et al., 2015). Similar to healthy adults, there seems to be no single unique scaling factor that eliminates sex differences for all parameters of the heart. Even when individuals are matched for age, height, and lean body mass, significant sex differences remain in the cardiovascular response to exercise (Charkoudian and Joyner, 2004).

### 3.3. Sex Differences in Athlete's Myocardial Strains

**The female athlete's heart has larger contractility than the male heart**. Table 5 summarizes the sex differences in strains and strain rates in athletes' hearts. In total, less than 150 cases of left ventricular strain in female athletes from two-dimensional speckle-tracking echocardiography have ever been published and most athletic disciplines remain untouched (Zacher et al., 2020). Interestingly, studies of healthy untrained people and elite athletes have shown that global strains do not change significantly with physiological adaptation to exercise, neither in women nor in men (Butz et al., 2011; Zacher et al., 2020). The general trend that the female heart has larger contractile strains than the male heart that we have observed for non-athletes in Table 2 persists for athletes in Table 5. For example, left ventricular longitudinal strains in female athletes, at -22, are +25% larger in absolute value than in male athletes, at -18% (Sanz-de la Garza et al., 2017). A study of male and female master's athletes confirmed this general trend, but with a lower percentage difference of only +7% (Wooten et al., 2020). Unfortunately, the data on sex differences in strains in athletes' hearts are very limited, the sample sizes are generally small, and it is difficult to draw robust conclusions.

**Table 5:**
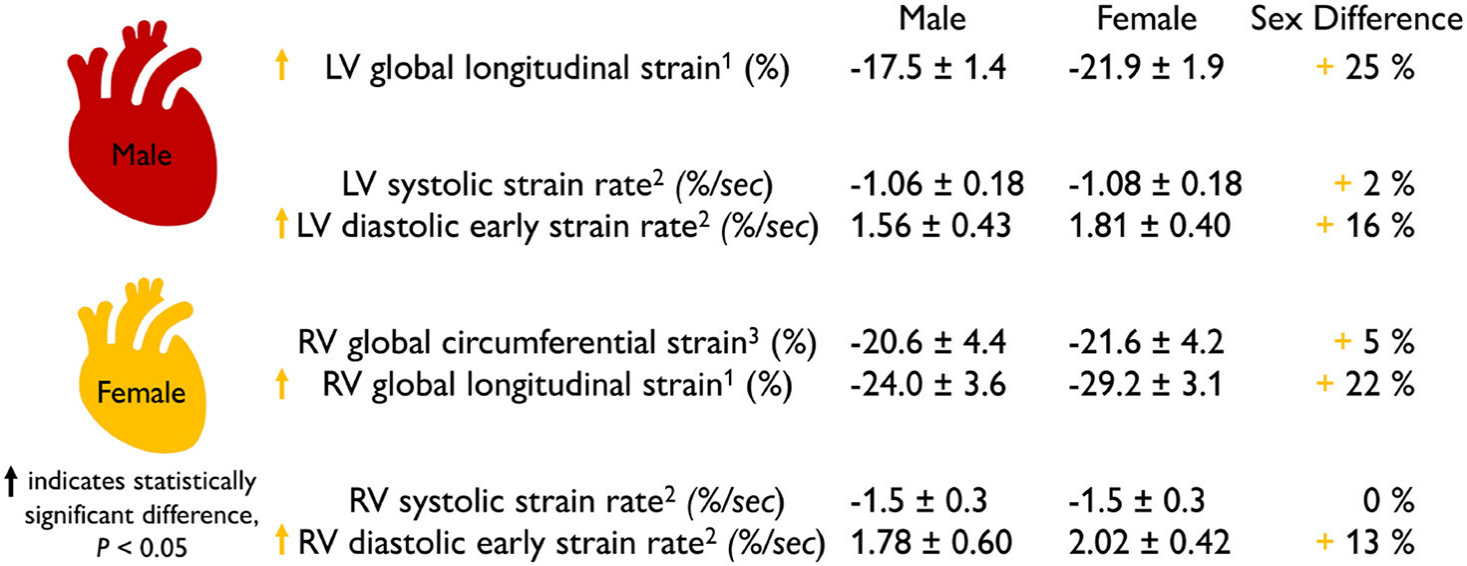
Sex differences in athlete's myocardial strains.

*The female athlete's heart has significantly greater strains than the male heart in the longitudinal axis for the left ventricle, and the circumferential and longitudinal axes for the right. Possibly due to small sample sizes, several strain parameters were not significantly different between the sexes. Negative values in red indicate that the female parameter is smaller; arrows indicate reported statistically significant differences; quantitative data are represented as mean absolute value ± SD; [1] Sanz-de la Garza et al. (2017) [2] Giraldeau et al. (2015) [3] Lakatos et al. (2018)*.

### 3.4. Sex Differences in Athlete's Blood Pressure

**The female blood pressure in athletes is lower than the male blood pressure**. Table 6 summarizes the sex differences in blood pressure for both male and female athletes' hearts using classical and modified cuff measurements. Unfortunately, studies on cardiac pressure in athletes are rare and a distinction between male and female athletes is even less common. The few available studies confirm the trend for non-athletes in Table 3 that female athletes have a slightly lower blood pressure than male athletes with differences on the order of -5% (Boraita et al., 2016; Tomschi et al., 2021). Blood pressure is often considered a driver for cardiac wall thickening. Notably, the reported septal wall thicknesses of male and female athletes of 10.1 and 8.7 mm (D'Ascenzi et al., 2020) are +10 and +6% larger than those of non-athletes with 9.2 and 8.2 mm (Vasan et al., 1997). An interesting question to ask is whether elevated blood pressure in athletes translates into thickening of the ventricular walls, and to which extent this adaptation differs by the type of exercise and by sex.

**Table 6:**
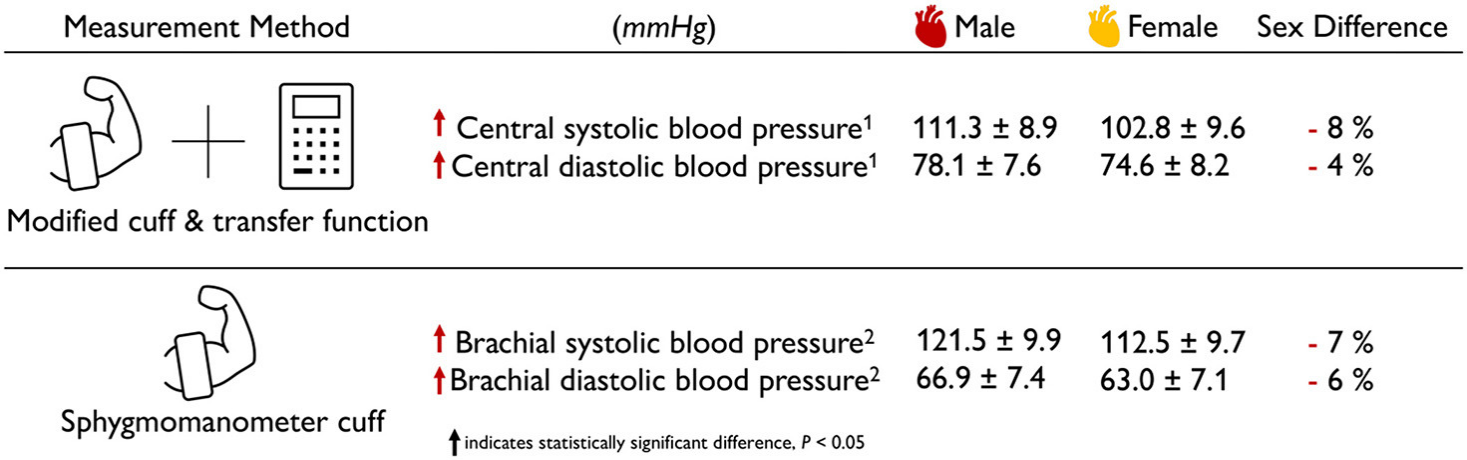
Sex differences in athlete's blood pressure.

*The female athlete's heart has lower blood pressure than the male heart when measured with standard non-invasive techniques using the blood pressure cuff. Negative values in red indicate that the female parameter is smaller; arrows indicate reported statistically significant differences; quantitative data are represented as mean absolute value ± SD; [1] Tomschi et al. (2021) [2] Boraita et al. (2016)*.

## 4. Physiological Adaptation in the Athlete's Heart

The human heart not only adapts its form and function to kinematic, hormonal, and genetic cues but also athletic exercise, both short- and long-term. In Section 3 on athlete's hearts, we have discussed *how* the human heart adapts to intense athletic exercise; in this Section, we discuss *why*. First, we review the general hypotheses and classification schemes for exercise-induced physiological cardiac growth and remodeling. Then, we discuss sex differences of cardiac adaptation in athletes.

### 4.1. Classification of Physiological Adaptation

**Figure 3:**
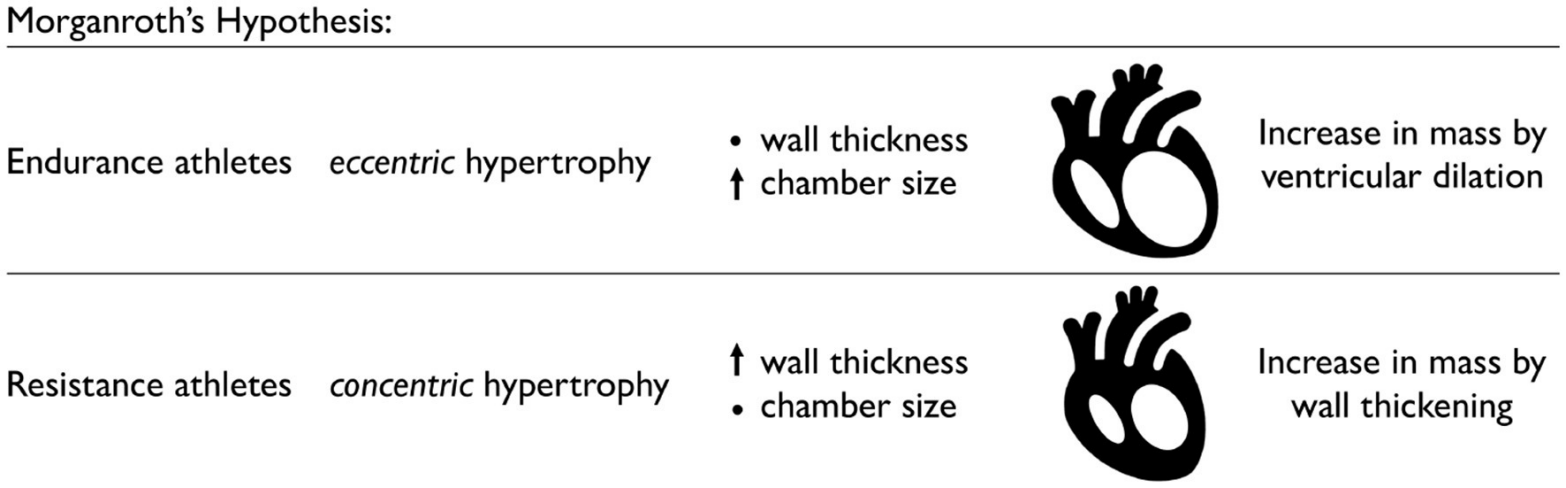
Physiological adaptation in endurance and resistance athletes. According to Morganroth's hypothesis, endurance athletes tend to develop eccentric hypertrophy associated with ventricular dilation caused by volume overload, while the wall thickness remains relatively constant. Resistance athletes tend to develop concentric hypertrophy associated with ventricular wall thickening caused by pressure overload, while the chamber size remains relatively constant.

**Endurance athletes develop ventricular dilation, while resistance athletes develop wall thickening**. Figure 3 visualizes Morganroth's hypothesis, a popular and commonly accepted paradigm in exercise physiology to characterize the effects of endurance and resistance training. Morganroth's hypothesis postulates that endurance athletes tend to develop eccentric hypertrophy associated with ventricular dilation caused by volume overload, while resistance athletes tend to develop concentric hypertrophy associated with ventricular wall thickening caused by pressure overload (Morganroth et al., 1975). For almost half a decade, this simple and easy-to-understand idea has shaped our understanding of how an athlete's heart adapts to physiological overload. Throughout the past decade, however, it has become increasingly clear that Morganroth's observations are an oversimplification of the underlying cardiac mechanics during exercise. The hypothesis is generally supported for endurance athletes but not for resistance athletes (Galderisi et al., 2015; Haykowsky et al., 2018; Kooreman et al., 2019). Both endurance and resistance athletes have hearts with a balanced increase in the chamber and wall dimensions (Galderisi et al., 2015; Kooreman et al., 2019). However, in contrast to Morganroth's initial hypothesis, endurance exercise is often associated with both pressure and volume overload (Haykowsky et al., 2018). At the same time, resistance athletes often do not develop wall thicknesses above the normal limits, while elite rowers, canoeists, and cyclists sometimes display abnormally large wall thicknesses (Spirito et al., 1994). To complicate matters, studies of concentric hypertrophy in resistance athletes can be confounded by the use of anabolic steroids, which tend to promote concentric hypertrophy (Haykowsky et al., 2018). An interesting question to ask is to which extent the physiological mechanisms that underpin Morganroth's hypothesis are related to a transient increase in preload and afterload during endurance and resistance training.

**Figure 4:**
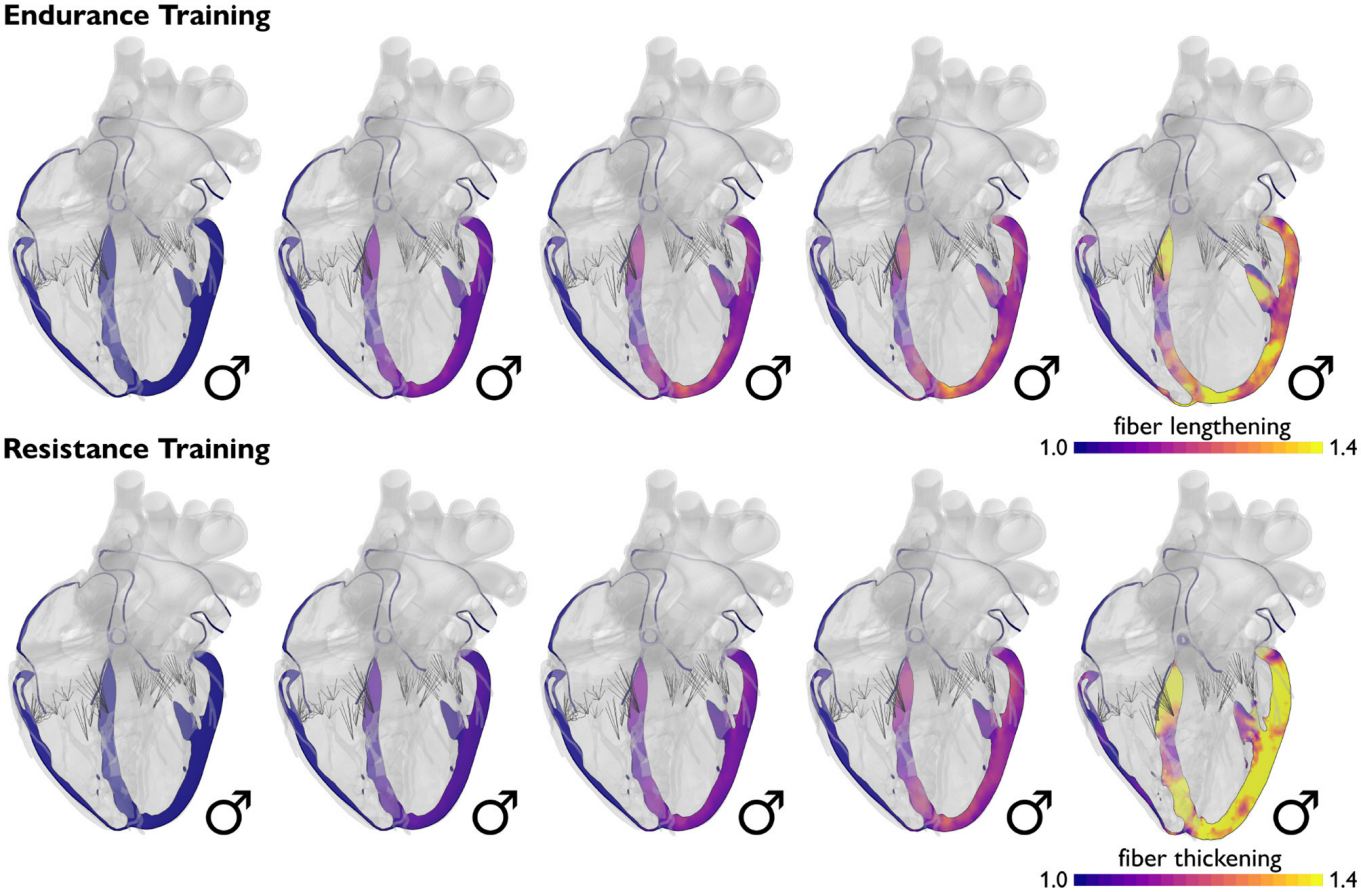
Physiological adaptation to volume and pressure overload. Volume overload induced by endurance training causes primarily eccentric growth associated with ventricular dilation, while pressure overload induced by resistance training causes primarily concentric growth associated with ventricular wall thickening. Simulations allow us to probe the effects of endurance and resistance training on muscle fiber length and thickness and on the geometry of the heart as a whole. Blue colors indicate the baseline fiber length and thickness; yellow colors indicate a fiber lengthening and thickening of up to 40%.

**Volume overload causes ventricular dilation, while pressure overload causes wall thickening**. Figure 4 illustrates how volume overload, primarily associated with endurance training, causes eccentric hypertrophy resulting in ventricular dilation, while pressure overload, historically associated with resistance training, causes concentric hypertrophy resulting in ventricular wall thickening (Genet et al., 2016). In practice, it is difficult to separate volume and pressure overload induced by endurance and resistance training. Computational simulations can provide a window into the individual effects of volume and pressure overload and predict molecular, cellular, and tissue level events that result in geometric and functional adaptation of an athlete's heart (Göktepe et al., 2010a). For the example in Figure 4, cardiac muscle fibers lengthen and thicken up to 40% (Peirlinck et al., 2021b). Increasing the amount of volume or pressure overload increases cardiomyocyte lengthening and thickening, which, in turn, results in larger and thicker ventricles. A natural benefit of computational modeling is that it allows us to probe and predict the effects of either type of overload and both types of overload combined (Göktepe et al., 2010b).

**Figure 5:**
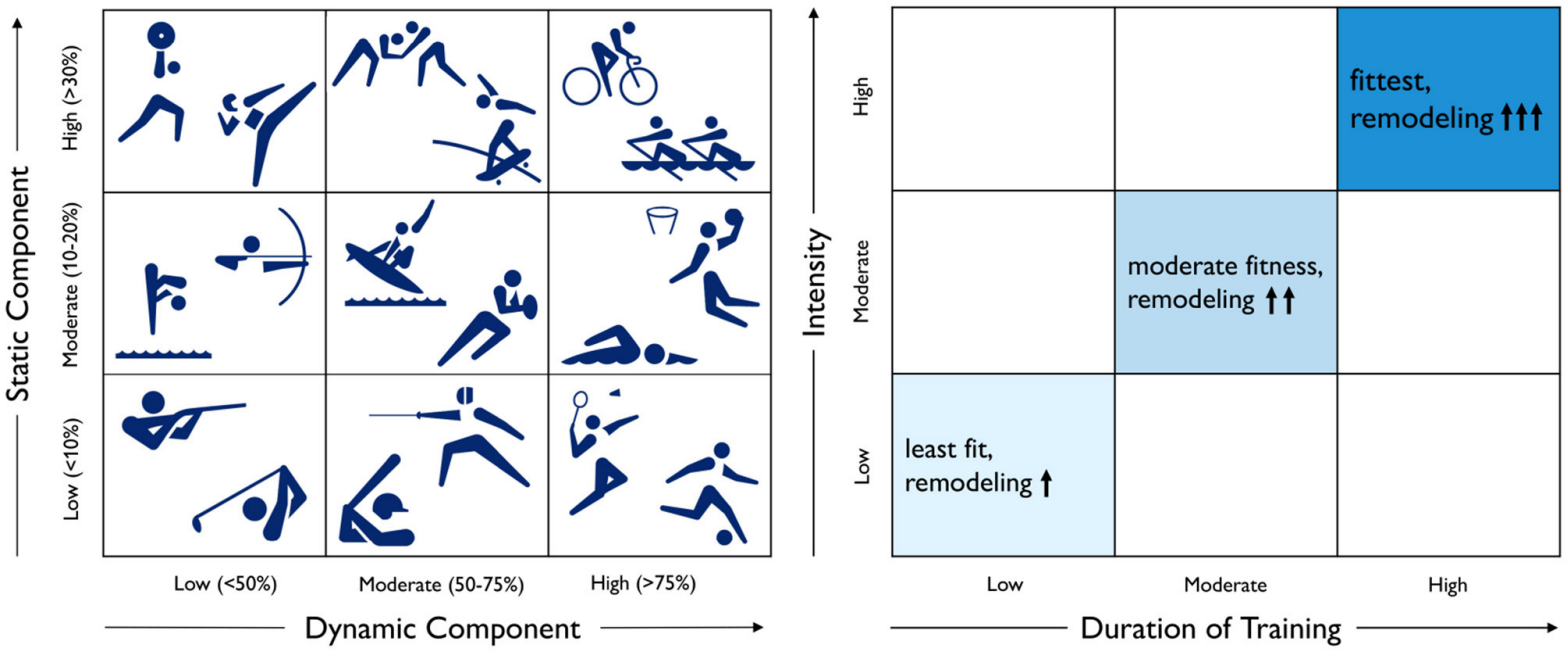
Physiological adaptation to dynamic and static exercise and training duration and intensity. According to Mitchell's classifications, left, dynamic exercise is associated with an increase in cardiac output, while static exercise is associated with an increase in blood pressure. According to Beaudry's classifications, right, the degree of cardiac adaptation depends on the duration and intensity of training.

**Dynamic exercise causes ventricular dilation, while static exercise causes wall thickening**. Figure 5, left, illustrates Mitchell's classifications, a common paradigm to categorize sports by their relative components of dynamic and static exercise (Mitchell et al., 2005). We can picture Mitchell's classifications for different athletic activities on a 3 × 3 grid based on their dynamic and static components (Levine et al., 2015; Beaudry et al., 2016). The underlying idea is that an increase in dynamic exercise is associated with an increase in cardiac output and an overload in volume, while an increase in static exercise is associated with an increase in blood pressure and an overload in pressure (Mitchell et al., 2005). While this classification scheme has been used successfully to predict cardiac adaptation based on the position of a sport in the grid, Mitchell's classification alone may not be the best method to estimate adaptation. It ignores the intensity and duration of training, as well as the variation among player positions for a given sport. For example, intense but short strength training often results in relatively little cardiac adaptation. This motivates an additional classification scheme that accounts for the duration and intensity of training (Beaudry et al., 2016).

**Duration and intensity of training increase the degree of adaptation**. Figure 5, right, illustrates Beaudry's classifications, a recent paradigm to categorize the impact of training on cardiac remodeling (Beaudry et al., 2016). We can picture Beaudry's classifications on a 3 × 3 grid based on the duration and intensity of training as an indicator for the level of fitness and the degree of cardiac remodeling. It would be straightforward to include these considerations into the computational model of Figure 4 to predict increased ventricular dilation or wall thickening with increased duration and intensity of endurance or resistance training (Peirlinck et al., 2021b).

### 4.2. Sex Differences in Physiological Adaptation

**Exercise does not change intrinsic sex differences in geometry and function**. Table 7 compares the sex differences in healthy and athletes' hearts on the basis of Tables 1, 2. Strikingly, the first and second columns suggest that the relative differences between female and male hearts do not change with exercise. Sex differences are the same in healthy and athlete's hearts across all geometric and functional features: the female heart is consistently smaller in mass, ventricular volumes, and cardiac output and larger in ejection fraction and heart rate. This seems to suggest that intense athletic exercise does not change intrinsic sex differences. The third and fourth columns compare the adaptation from healthy to athlete for both men and women, with the healthy heart considered as baseline. Both male and female hearts are capable of adapting their form and function to athletic exercise with the largest increases, from 37.4% to 136%, in the left and right ventricular volumes. Unexpectedly, the right ventricular mass, ejection fraction, heart rate, and cardiac output in Table 7 decreased for both men and women. It is unclear whether this is caused by physiological differences or differences in measurement techniques between the different studies. All other observations are in line with several studies that reported no sex-specific adaptation to exercise in functional or structural cardiac parameters (Petersen et al., 2006). It would be interesting to understand whether and how the reported intrinsic sex differences on the global whole organ scale, independent of the exercise level, translate into differences on the local tissue and cellular scale.

**Table 7:**
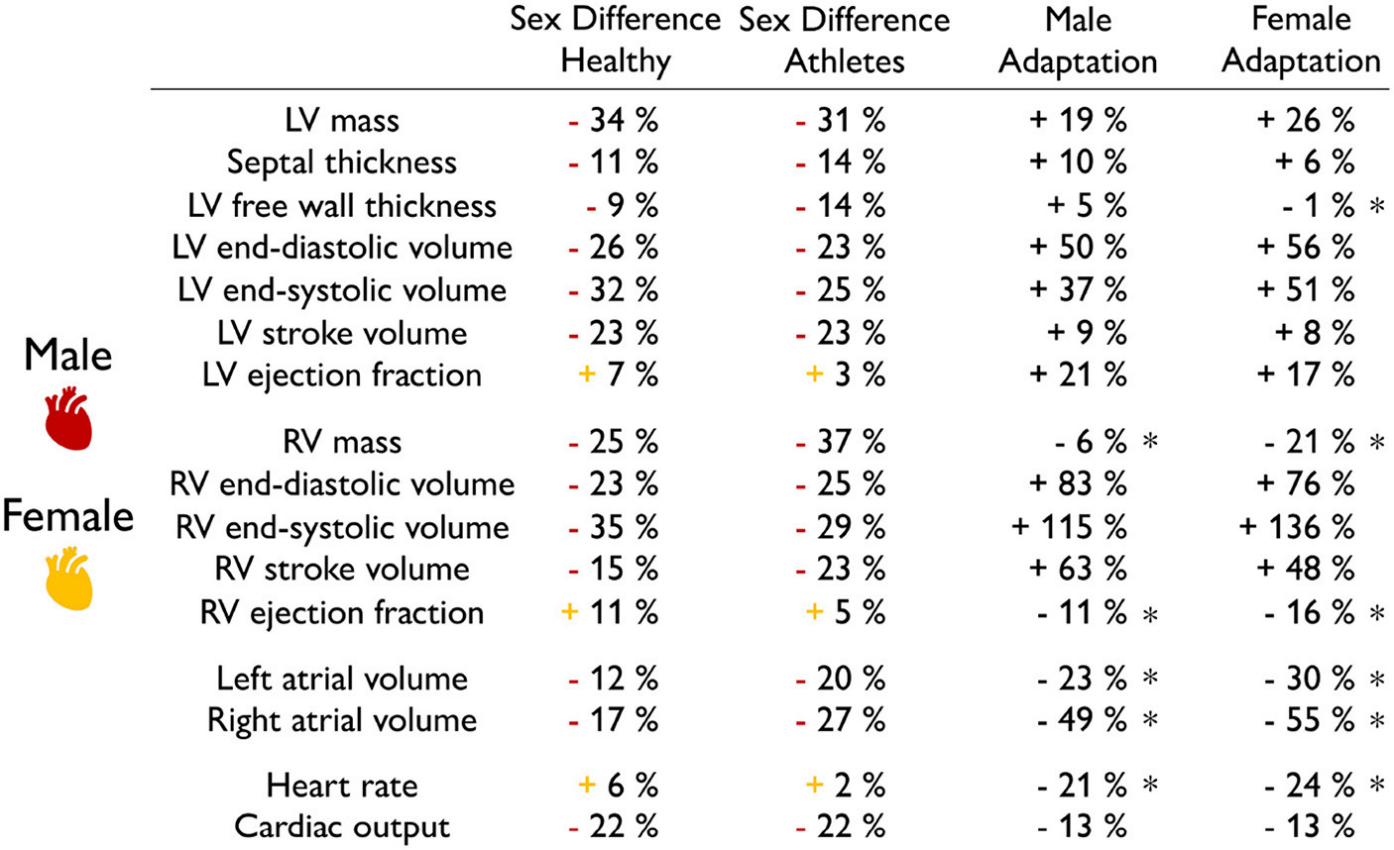
Sex differences in physiological adaptation.

Sex differences between female and male healthy non-athletes and athletes are comparable in magnitude across all geometric and functional features suggesting that exercise does not change intrinsic sex differences. Adaptation from health to athlete's hearts reveals significant changes with similar trends in men and women. Percentage differences between male and female for healthy and athlete's hearts, and percentage adaptation from healthy to athlete for male and female hearts; ^*^ indicates an unexpected decrease that could, in part, be caused by differences in measuring techniques across studies. Negative values in red indicate that the female parameter is smaller.

**Figure 6:**
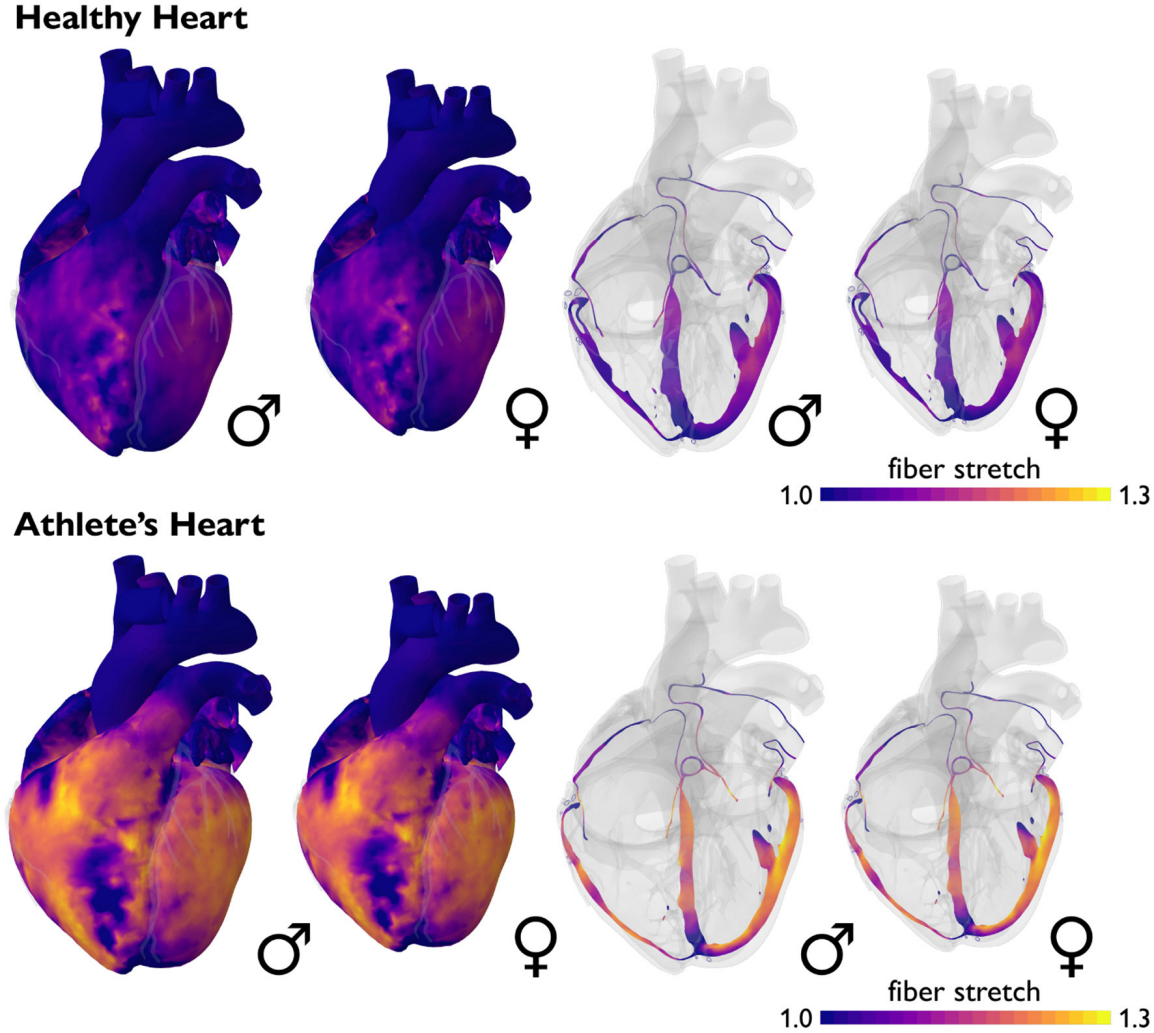
Sex differences in short-term fiber stretch and elastic response. Simulated short-term fiber stretch for male and female hearts, left and right, subject to the stroke volumes of healthy and athlete's hearts, top and bottom. In the healthy heart, simulated fiber stretches remain well within the physiological regime of 1.10 to 1.15, with a few local peaks on the order of 1.20. In the athlete's heart, simulated fiber stretches would exceed the physiological regime and reach values of 1.20 to 1.30, with almost the entire heart experiencing stretches above 1.20. Volume overload on the whole heart level induces an overstretch on the cellular level and triggers physiological adaptation in the athlete's heart. Male and female hearts respond similarly under both healthy and athletic conditions.

**Male and female hearts respond similarly to both healthy and athletic conditions**. Figure 6 illustrates the simulated short-term fiber stretch for male and female hearts, left and right, subject to the stroke volumes of healthy and athlete's hearts, top and bottom. The model uses isogeometrically scaled male and female heart geometries to predict the physiological short-term response of an average male and female endurance athlete's heart. As initial conditions, we use the healthy male and female heart geometries according to Figure 2. We gradually load both models with the stroke volumes of healthy and athlete's hearts from Tables 1, 4 and simulate the purely elastic response. For the healthy heart simulation, we fill the male left and right ventricles with their baseline stroke volumes, and decrease the stroke volume in the female left and right ventricles by −23 (Rutkowski et al., 2020) and −15% (Tandri et al., 2006) compared to baseline. For the athlete's heart simulation, we increase the stroke volume in the male left and right ventricles by +9 (Giraldeau et al., 2015) and +63% (Csecs et al., 2020) and in the female left and right ventricles by +8 (Giraldeau et al., 2015) and +48% (Csecs et al., 2020) compared to the healthy baseline. In a healthy heart, these stroke volumes would induce fiber stretches that remain well within the physiological regime of 1.10 to 1.15, with a few local peaks on the order of 1.20. In the athlete's heart, these stroke volumes would induce severely elevated fiber stretches on the order of 1.20 to 1.30, with almost the entire heart experiencing stretches above 1.20. These values significantly exceed the physiological regime and imply that the myocardium would no longer be able to optimally contract (Gordon et al., 1966; Shiels and White, 2008). This simulation illustrates why the athletes' heart has to adapt in size to compensate for larger stroke volumes. In other words, stroke volumes observed in athlete's hearts are only possible if the heart adapts to volume overload. This is indeed what Morganroth's hypothesis suggests (Morganroth et al., 1975) and what the numbers in Tables 1, 4 confirm. Notably, in contrast to the strain differences in Tables 2, 5, the simulation predicts similar stretch profiles for the male and female hearts under both healthy and athletic conditions. This could point toward a limitation of our model, which uses isogeometrically scaled male and female heart geometries and ignores the effect of disproportionally smaller left ventricles in female hearts.

**Figure 7:**
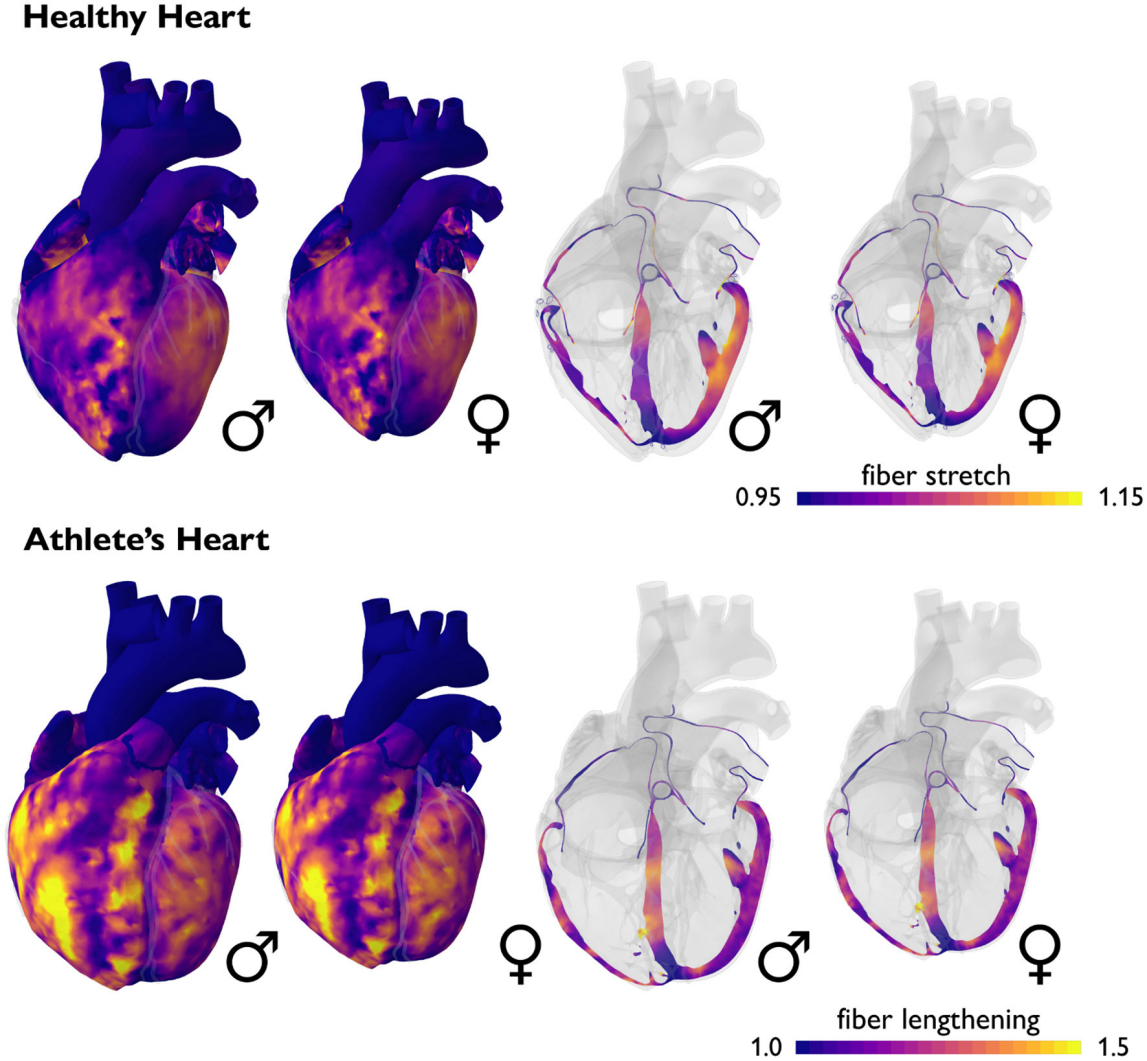
Sex differences in long-term fiber lengthening and adaptive growth. Simulated long-term fiber stretch for male and female hearts, left and right, subject to the stroke volumes of healthy and athlete's hearts, top and bottom. In the healthy heart, fiber stretches remain well within the physiological regime of 1.10 to 1.15, with a few local peaks on the order of 1.20. In the athlete's heart, chronically elevated fiber stretches trigger a chronic fiber lengthening of up to 1.50. Volume overload on the whole heart level induces an overstretch on the cellular level and triggers physiological adaptation in the athlete's heart. Male and female hearts respond similarly under both healthy and athletic conditions.

**Male and female hearts adapt similarly under both healthy and athletic conditions**. Figure 7 illustrates the simulated long-term fiber stretch and fiber lengthening for male and female hearts, left and right, subject to the stroke volumes of healthy and athlete's hearts, top and bottom. The model uses the same heart geometries and stroke volumes for male and female healthy and athlete's hearts as in the previous example, however, now, we perform a long-term simulation during which the heart is allowed to gradually adapt (Peirlinck et al., 2019). We have seen in Figure 6 that an exercise-induced chronic volume overload in athletes increases the diastolic fiber stretch. This triggers the addition of sarcomeres in series and a relative increase in cardiomyocyte length associated with eccentric hypertrophy and ventricular dilation (Göktepe et al., 2010a). This physiological adaptation allows both male and female athlete's hearts to relax to similar homeostatic stretch states as in the male and female healthy hearts. Similar to the generic growth model in Figure 4, the sex-specific volume overload in male and female hearts in Figure 7 manifests itself in complex heterogeneous three-dimensional growth patterns. Similar to the short-term elastic simulation in Figure 6, the long-term growth simulation predicts similar fiber lengthening profiles for the male and female hearts. The simulation highlights the need for more realistic female heart models. Realistic image-based female heart geometries, that are not just purely based on isometric scaling, but account for sex-specific differences in geometric features, are critical to making accurate predictions and differentiating between physiological and pathological adaptation in female hearts.

## 5. Discussion

**Current diagnostic criteria for enlarged hearts are not sex-specific**. Cardiomyopathies cause the heart to adapt in a pathological manner and result in reduced function. Comparable to endurance and resistance training, dilated cardiomyopathy is associated with volume overload, eccentric growth, and ventricular dilation, while hypertrophic cardiomyopathy is associated with pressure overload, concentric growth, and ventricular wall thickening (Göktepe et al., 2010b). Standard and widely accepted diagnostic criteria are a left ventricular ejection fraction of less than 50% for dilated cardiomyopathy (Cannatà et al., 2020) and a ventricular wall thickness greater than 15 mm for hypertrophic cardiomyopathy (Gersh et al., 2011). Notably, the diagnostic criteria for cardiomyopathies are generally not sex-specific.

**Prevalence, age at diagnosis, and severity of symptoms of cardiomyopathies are sex-specific**. Table 8 summarizes the sex differences in prevalence, age at diagnosis, and severity of symptoms in both dilated and hypertrophic cardiomyopathies. On the basis of non-sex-specific diagnostic criteria, the male-to-female ratio in the prevalence of dilated and hypertrophic cardiomyopathies is 3:1 and 3:2, suggesting that men are more likely to be diagnosed with cardiomyopathies than women (Olivotto et al., 2005; Cannatà et al., 2020). Men are twice as likely as women to be diagnosed with hypertrophic cardiomyopathy by routine exam (Olivotto et al., 2005). Interestingly, men are diagnosed earlier and with less severe symptoms than women (Olivotto et al., 2005; Halliday et al., 2018; Cannatà et al., 2020). Taken together, these findings emphasize the critical need for sex-specific criteria to diagnose dilated and hypertrophic cardiomyopathies, early, robustly, and reliably.

**Table 8:**
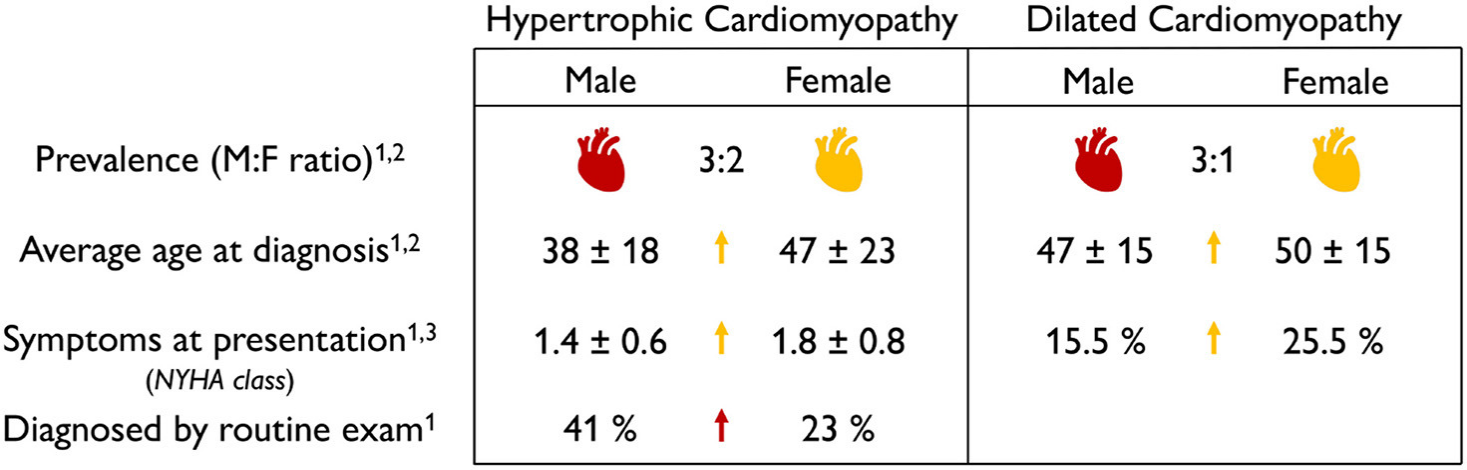
Sex differences in pathological adaptation.

*Women are diagnosed with dilated and hypertrophic cardiomyopathies at older ages and with greater symptoms at presentation than men. Men are twice as likely to be diagnosed with hypertrophic cardiomyopathy by a routine exam than women. [1] Olivotto et al. (2005) [2] Cannatà et al. (2020) [3] Halliday et al. (2018)*.

**Figure 8:**
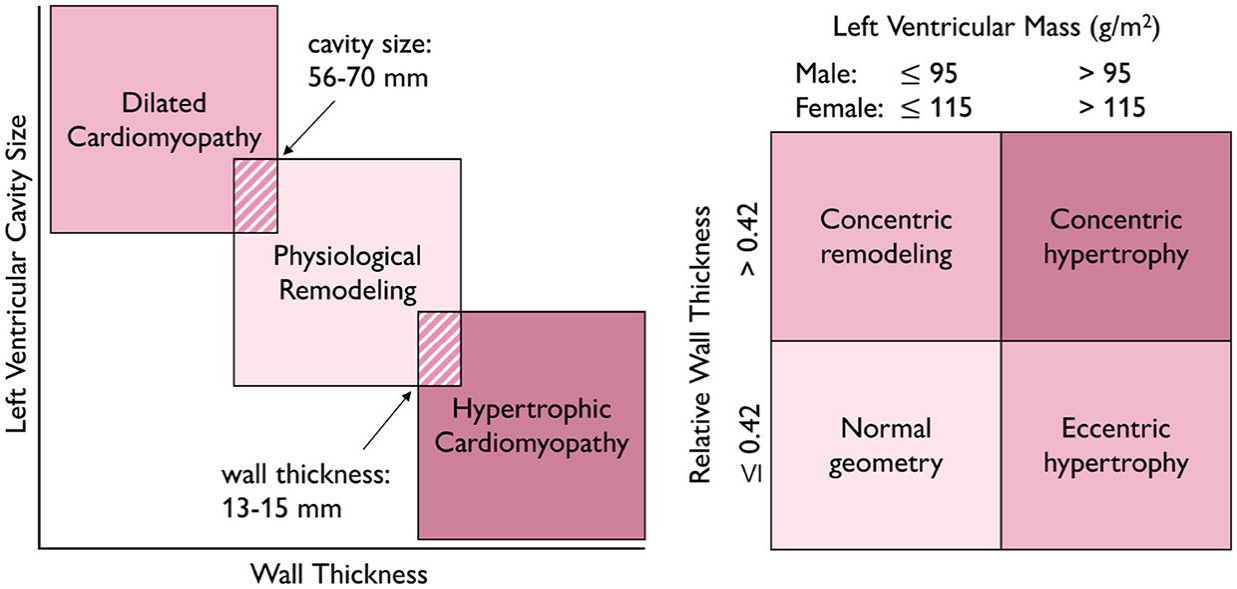
Differentiating physiological from pathological adaptation. Wall thickness and cavity size are diagnostic criteria for which a subset of physiologically remodeled athlete's hearts overlaps with pathologically remodeled dilated and hypertrophic cardiomyopathy hearts, left. Relative wall thickness and sex-specific left ventricular mass are criteria which differentiate normal geometry from hypertrophic, even in the case of extreme athletic adaptation, right.

**Current diagnostic criteria fail to discriminate physiological and pathological adaptation**. Figure 8 summarizes four potential criteria to differentiate physiological from pathological adaptation: wall thickness, left ventricular cavity size, sex-specific left ventricular mass, and relative wall thickness. Discriminating physiological remodeling in athletes' hearts from pathological remodeling in dilated and hypertrophic cardiomyopathy remains a major challenge for physicians. When using just the wall thickness and left ventricular cavity size, a subset of physiologically remodeled athlete's hearts would overlap with pathologically remodeled dilated and hypertrophic cardiomyopathy hearts, refer to Figure 8, left: between 1–8% of female athletes have a left ventricular cavity size of 56–70 mm, which would classify them as having dilated cardiomyopathy (Dewey et al., 2008). Approximately 1.7–2.5% of male athletes have a left ventricular wall thickness of 13–15 mm, which would classify them as having hypertrophic cardiomyopathy (Dewey et al., 2008). In general, female athletes are more likely to have dilated ventricles, while male athletes are more likely to have thick ventricular walls (Dewey et al., 2008). Relative wall thickness, the ratio between wall thickness and ventricular diameter, could be a more specific criterion than absolute wall thickness when distinguishing normal from hypertrophied hearts in both female and male athletes (Finocchiaro et al., 2017), refer to Figure 8, right.

**Diagnostic criteria for cardiomyopathies should be sex-specific**. The lack of sex-specific diagnostic criteria for cardiomyopathies has a significant impact on women. As we have discussed, the female heart is neither the same as the male heart, nor is it simply a small version of it. The 15 mm maximum wall thickness threshold for hypertrophic cardiomyopathy in female hearts implies that, from a purely geometric standpoint, the female heart will already have a much larger degree of hypertrophy than the male heart by the time it is diagnosed (van Driel et al., 2019). Using sex-specific assays to diagnose type 1 myocardial infarction results in 30% more women and 4.9% more men being diagnosed, with all patients exhibiting the same symptoms associated with this pathology (Ferry et al., 2019). Although fewer women are diagnosed with cardiovascular disease than men, these women are more likely to die from the disease (Sobhani et al., 2018). The use of sex-specific biomarkers is critical to address and ultimately reduce the mortality gap between women and men in a cardiovascular disease (Sobhani et al., 2018). A multimodal computational framework that integrates hemodynamics, medical images, patient history, and modeling could provide the basis to develop sex-specific diagnostic criteria for cardiomyopathies. For example, this approach could use statistical shape modeling to represent variations in healthy and diseased populations–separated by sex–and use machine learning to identify classifiers like wall thickness, diameter, and volume, and systematically correlate them with sex-specific disease outcomes.

**Diagnostic criteria for heart failure should use sex-specific ejection fractions**. Heart failure is currently diagnosed with sex-neutral ejection fraction thresholds (Chung et al., 2006). For example, the criterion for heart failure with preserved left ventricular ejection fraction is ≥ 50% (Ponikowski et al., 2016). However, women have a higher baseline ejection fraction than men. When the same ejection fraction criteria are used for both female and male patients, this may under-diagnose women whose baseline ejection fraction is higher then that of men, refer to Table 1. Ideally, for the diagnosis of heart failure with preserved ejection fraction, the critical ejection fraction threshold in women should be much higher (Kaila et al., 2012). This recommendation is supported by studies according to which women benefit from therapies at a higher range of left ventricle ejection fractions than used for men (McMurray et al., 2020; Solomon and McMurray, 2021). Clearly, sex-specific research is necessary to understand how heart failure impacts women and men differently (Lala et al., 2021), and data-driven modeling could play a central role to advance more accurate diagnostics in this field.

**Computational modeling provides a window into sex differences between female and male hearts**. Understanding the effects of sex differences in hormones, genes, cells, tissue, and the heart as a whole is a complex endeavor. Numerous data sources are available to characterize the role of sex as a biological variable in healthy, athlete's, and diseased hearts. However, integrating multimodal, multiscale data into simple sex-specific diagnostic criteria remains a challenging task (Peirlinck et al., 2021a). As a solution to these issues, data-driven computational modeling offers a compelling framework to integrate detailed geometrical, functional, and microstructural information in a consistent and objective manner (Trayanova and Rice, 2011; Lopez-Perez et al., 2015; Peirlinck et al., 2021b). For example, using a hybrid experimental-computational approach, a recent study has shown that women are at a higher risk for Torsades de Pointes than men (Yang et al., 2017). Increasing progesterone in women and testosterone in men can reduce arrhythmic risk. Sex-specific cardiomyocyte models have also been used to develop sex-specific classification schemes for Torsades de Pointes in response to different drugs both on the cellular level (Fogli Iseppe et al., 2021) and on the whole heart level (Peirlinck et al., 2021a). Using the basic laws of physics, combined with clinical and experimental data from various sources, we can integrate finite element analysis, similar to Figures 4, 6, 7, and machine learning (Peirlinck et al., 2021c) to estimate important cardiac features that are out of reach for current measurement techniques today. The resulting computational tools provide a powerful platform to test the sex-specific hypotheses that have been proposed throughout this review. For example, if the female heart is truly made up of one-fifth more ventricular cardiomyocytes than the male heart (Litviňuková et al., 2020), this could have serious implications on the constitutive models to simulate the healthy heart. Classical constitutive models (Holzapfel and Ogden, 2009) were originally based on porcine hearts (Dokos et al., 2002) and more recently supplemented by human tissue samples (Sommer et al., 2015). Yet, none of these authors explicitly distinguished between female and male hearts.

## 6. Conclusion

The female heart is not only one-fourth smaller than the male heart, but it also has a different microstructural architecture. It has a larger ejection fraction and beats at a faster rate but generates a smaller cardiac output. It has a lower blood pressure but produces universally larger contractile strains. Allometric scaling, for example by lean body mass, can reduce but not eliminate the sex differences between female and male hearts. Importantly, our review demonstrates that sex differences between female and male hearts are too complex to be ignored: The female heart is not just a small version of the male heart. When using similar diagnostic criteria for female and male hearts, cardiac disease in women is frequently overlooked by routine exams; it is diagnosed later and with more severe symptoms than in men. Clearly, there is an urgent need to better understand the female heart and design sex-specific diagnostic criteria that will allow us to diagnose cardiac disease in women equally as early, robustly, and reliably as in men.

## Data Availability Statement

The original contributions presented in the study are included in the article/supplementary files, further inquiries can be directed to the corresponding author.

## Author Contributions

SS performed the literature review, created the illustrations, and wrote the manuscript. MP performed the simulations and wrote the manuscript. EK helped design the study and wrote the manuscript.

## Funding

This research was part of the Digital Athlete Project within the Wu Tsai Human Performance Alliance. It is inspired by the Dassault Systèmes Living Heart Project and supported by the National Science Foundation Graduate Research Fellowship to SS, by a Belgian American Education Foundation Research Fellowship to MP, and by the NIH grant 5R01HL131823 to EK.

## Conflict of Interest

The authors declare that the research was conducted in the absence of any commercial or financial relationships that could be construed as a potential conflict of interest.

## Publisher's Note

All claims expressed in this article are solely those of the authors and do not necessarily represent those of their affiliated organizations, or those of the publisher, the editors and the reviewers. Any product that may be evaluated in this article, or claim that may be made by its manufacturer, is not guaranteed or endorsed by the publisher.
